# Comparison of antipsychotic drug use in children and adolescents in the Netherlands before and during the COVID-19 pandemic

**DOI:** 10.1007/s00787-023-02340-3

**Published:** 2024-01-06

**Authors:** Ravish N. Gangapersad, Guiling Zhou, Pilar Garcia-Gomez, Jens Bos, Eelko Hak, Birgit C. P. Koch, Catharina C. M. Schuiling-Veninga, Bram Dierckx

**Affiliations:** 1 Department of Hospital Pharmacy, Erasmus University Medical Center, Rotterdam, The Netherlands; 2 Erasmus School of Economics, Erasmus University Rotterdam, Rotterdam, The Netherlands; 3 Unit of Pharmaco-Therapy, -Epidemiology and -Economics (PTEE), Department of Pharmacy, University of Groningen, Groningen, The Netherlands; 4 Department of Child and Adolescent Psychiatry/Psychology, Erasmus Medical Center, Rotterdam, The Netherlands

**Keywords:** COVID-19, Antipsychotics, Prevalence, Incidence, ARIMA

## Abstract

This study aims to describe the patterns and trends in antipsychotic prescription among Dutch youth before and during the corona virus disease 2019 (COVID-19) pandemic (between 2017 and 2022). The study specifically aims to determine whether there has been an increase or decrease in antipsychotic prescription among this population, and whether there are any differences in prescription patterns among different age and sex groups. The study utilized the IADB database, which is a pharmacy prescription database containing dispensing data from approximately 120 community pharmacies in the Netherlands, to analyze the monthly prevalence and incidence rates of antipsychotic prescription among Dutch youth before and during the pandemic. The study also examined the prescribing patterns of the five most commonly used antipsychotics and conducted an autoregressive integrated moving average (ARIMA) analysis using data prior to the pandemic, to predict the expected prevalence rate during the pandemic. The prescription rate of antipsychotics for Dutch youth was slightly affected by the pandemic, with a monthly prevalence of 4.56 [4.50–4.62] per 1000 youths before COVID-19 pandemic and 4.64 [4.59–4.69] during the pandemic. A significant increase in prevalence was observed among adolescent girls aged 13–19 years. The monthly incidence rate remained stable overall, but rose for adolescent girls aged 13–19 years. Aripiprazole, and Quetiapine had higher monthly prevalence rates during the pandemic, while Risperidone and Pipamperon had lower rates. Similarly, the monthly incidence rates of Aripiprazole and Olanzapine went up, while Risperidone went down. Furthermore, the results from the ARIMA analysis revealed that despite the pandemic, the monthly prevalence rate of antipsychotic prescription was within expectation. The findings of this study suggest that there has been a moderate increase in antipsychotic prescription among Dutch youth during the COVID-19 pandemic, particularly in adolescent females aged 13–19 years. However, the study also suggests that factors beyond the pandemic may be contributing to the rise in antipsychotic prescription in Dutch youth.

**Supplementary Information:**

The online version contains supplementary material available at 10.1007/s00787-023-02340-3.

## Introduction

The coronavirus disease 2019 (COVID-19) pandemic has had significant impacts on the provision of healthcare services and the mental health of young people worldwide. Outpatient appointments were reduced or postponed, admissions to emergency departments were limited, and face-to-face services were less available during the pandemic [1, 2]. As a result, there are indications that limited access to healthcare may have led to a decrease in psychotropic medication prevalence. In a study done in the US, it was found that antidepressants declined by 7.5%, anxiolytics by 5.6%, and antipsychotics by 2.6% compared to expected levels across all age groups [3]. However, results from the UK are conflicting, with some studies suggesting a possible reduction in antipsychotics among children and adolescents accessing mental health services, while others report an increase in all psychotropic medications [4, 5]. In the Netherlands, there is evidence that the number of physical consultations at both general practitioners and emergency care decreased [6]. At the same time, however, many health care providers tried to combat this problem by providing e-health solutions. Indeed, a survey on e-health use in the Netherlands showed that 75% of the practices that responded had adopted new e-health applications since the start of the pandemic [7].

Moreover, the pandemic had a significant impact on the mental health of children and adolescents, with increased levels of depression, anxiety, and psychological distress. Several studies indicate that teenage females and high school seniors experienced the most significant decline in mental health [8]. For example, anxiety symptoms ranged from 1.8% to 23.87% in school-aged children (7–13 years) and from 10.4% to 29.27% in adolescents. There was also an increase in the reported aggression in all age groups. The mental health of children and adolescents in the Netherlands also worsened during the pandemic due to lockdowns, with an increase in severe anxiety and sleep-related impairment [9].

The impact of the pandemic itself on the use of antipsychotic drugs among children and adolescents is unclear. Several factors may have influenced the prescribing patterns of psychotropic medications. On one hand, the limited availability of healthcare services may have reduced access to appropriate psychiatric treatment. On the other hand, the increased prevalence of mental health issues may have heightened the demand for psychiatric care. Delays in getting adequate support may put children and adolescents with emotional problems at risk of mental breakdown, self-harm, suicidal thoughts, or violent behavior. These situations may require the use of psychiatric medication to alleviate acute distress and manage behavioral problems. Antipsychotic drugs may be prescribed in cases of psychiatric emergencies where children or adolescents show severe symptoms such as aggression, psychosis, or acute agitation [10, 11].

Recent studies show an increase in antipsychotic medication prescriptions for children and adolescents in the US during the pandemic, particularly among adolescent girls [12]. Furthermore, a study from Denmark reported that psychotropic medication use increased during the COVID-19 pandemic compared to previous years, with children aged 12–17 years the most affected [13]. Before the pandemic, the prevalence of antipsychotic prescriptions in the Netherlands stabilized from 2005 to 2019, with increased awareness among medical professionals of the negative effects of antipsychotics on children being a contributing factor [14]. To the best of our knowledge there is no evidence about the impact of the COVID-19 pandemic on antipsychotic use in the Dutch youth.

This study aims to describe antipsychotic prescription patterns and trends among youth in the Netherlands before and throughout the COVID-19 pandemic (between 2017 and 2022). Specifically, we hypothesize that the pandemic-induced stress and the delayed access to mental healthcare services may have contributed to more psychiatric emergencies and increased antipsychotic prescriptions in this population, particularly for adolescents. Understanding changes in antipsychotic prescription patterns during the pandemic may help inform policies and interventions aimed at mitigating the mental health impact of the pandemic on children and adolescents in the Netherlands.

## Methods

### Study design and data source

The study aimed to evaluate the impact of the COVID-19 pandemic on antipsychotic drug utilization. Data were obtained from the IADB.nl pharmacy prescription database of the University of Groningen, which contains dispensing data from approximately 120 community pharmacies and covers over 1,120,000 individuals for more than 25 years in the northern region of the Netherlands. This region is more rural than the central area of the country, with a more homogenous ethnic composition. However, it also has some urban centers that offer a contrast to the rural landscape. The data include information on patient characteristics, prescribed drugs (classified by the Anatomical Therapeutic Chemical (ATC) classification), prescription date, daily dose, and number of drug units dispensed. In-hospital prescriptions and over-the-counter medications were not included in the data, and the data were anonymized to ensure confidentiality. Total population estimates were extracted from the Dutch Central Bureau for Statistics (CBS). The use of the data for research is according to the European general data protection regulation (GDPR).

### Study population

The study period covered a pre-pandemic period from January 2017 to February 2020 and the pandemic period from March 2020 to December 2022 (the first COVID-19 case in the Netherlands was diagnosed on February 27th 2020 and the first lockdown on the 23rd of March 2020). The prevalent users were defined as individuals aged 19 years and younger (0–19) who were dispensed an antipsychotic (ATC N05A) at least once from January 2017 to December 2022 and registered in the IADB database. For prevalent users, age was defined on the first of January each year. The incident users were defined as children who initiated the dispensing of the antipsychotic within the study period and had no corresponding dispensing 365 days before the index date. The index date was the date of the first dispensing during the study period. For incident users, age was defined on the first of January each year.

### Outcome measures

The primary outcomes were the monthly prevalence rate (dispensing rate) and the incidence rate, stratified by sex and age groups (0–6 years, 7–12 years, 13–19 years), which were calculated as the number of prevalent users or incident users in each month per 1000 IADB persons for each corresponding year, respectively. Furthermore, we looked at the distribution of the type of prescribers for incident users and the number of combined pharmacotherapy users per year for prevalent users. Combined pharmacotherapy was defined as the use of more than one antipsychotic concurrently for at least 90 days in the same period [15]. The IADB population estimate is annual, and the population is a dynamic stationary cohort throughout the year without substantial change in size and distribution of risk factors.

### Statistical analysis

We performed descriptive analyses of age and sex for the IADB population from 2017 to 2022 and summarized the annual baseline characteristics. We used mean and standard deviation (SD) for continuous variables and proportions for categorical variables. To analyze the interrupted time-series data and account for autocorrelation, seasonality, underlying trends, and the impact of the pandemic, we used an autoregressive integrated moving average (ARIMA) model. We also visualized the impact of the pandemic on prevalence and incidence of antipsychotic prescriptions by fitting an ARIMA model with only pre-pandemic monthly data from 1 January 2017 to 1 March 2020. This model was used to forecast the expected values after 1 March 2020 without the pandemic. We used the auto.arima() function in *R* to obtain the best-fit ARIMA model, combining unit root tests, minimization of Akaike’s information criteria (AIC), and maximum likelihood estimation (MLE). We examined the model fit by using the Ljung–Box test and Box–Pierce test to check for autocorrelation of the residuals. Furthermore, we calculated the Mean Absolute Percentage Error (MAPE), which measures the average percentage difference between the predicted values and the actual values, to assess the model fit on the unseen data. All analyses were performed using *R*, version 4.2.1, with a two-tailed significance threshold of 0.05 for all statistical tests. The mean and 95% confidence interval of each variable were also estimated. The *t*-test was used to compare the means.

## Results

The total population aged 0–19 years ranged from 266,835 persons in 2017 to 238,732 persons in 2022. Between 2017 and 2022, a total of 6339 youths aged 0–19 years present in the IADB database were prescribed antipsychotic drugs.

### Prevalence

**Fig. 1:**
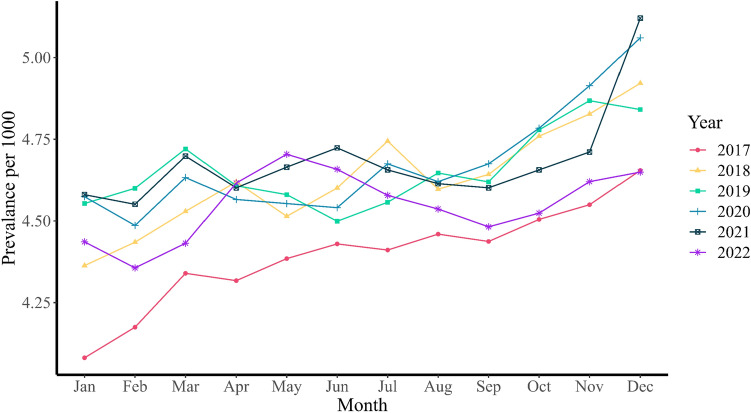
Monthly prevalence rate over the years

The average monthly prevalence rates of antipsychotic drug prescriptions were 4.56 (95% CI 4.50–4.62) pre-COVID-19 (Jan 2017–Feb 2020) and 4.64 (95% CI 4.59–4.69) per 1000 youths during COVID-19 (Mar 2020–Dec 2022). The prevalence rates stratified by age, sex and period are presented in Table 1 and the monthly prevalence rate stratified by calendar year can be seen in Fig. 1. The age-distribution of our population is presented in Supplementary Table 3.

**Table 1:** Average monthly prevalence (per 1000) of antipsychotic drug prescriptions among Dutch youth up to 19 years

	Pre-COVID-19 (Jan 2017–Feb 2020)	COVID-19 (Mar 2020–Dec 2022)	*P*-value
Total			
All ages	4.56 [4.50, 4.62]	4.64 [4.59, 4.69]	0.041
0–6	0.37 [0.34, 0.40]	0.25 [0.23, 0.28]	< 0.001
7–12	5.10 [5.00, 5.20]	4.80 [4.70, 4.90]	< 0.001
13–19	7.42 [7.33, 7.50]	7.85 [7.76, 7.94]	< 0.001
Males			
All ages	6.21 [6.16, 6.27]	5.90 [5.79, 6.01]	< 0.001
0–6	0.51 [0.48, 0.55]	0.42 [0.38, 0.46]	< 0.001
7–12	7.77 [7.62, 7.91]	7.03 [6.85, 7.21]	< 0.001
13–19	9.63 [9.56, 9.71]	9.40 [9.25, 9.54]	0.005
Females			
All ages	2.84 [2.75, 2.93]	3.34 [3.25, 3.43]	< 0.001
0–6	0.22 [0.19, 0.25]	0.08 [0.06, 0.09]	< 0.001
7–12	2.30 [2.24, 2.36]	2.47 [2.37, 2.57]	0.006
13–19	5.19 [4.99, 5.39]	6.32 [6.14, 6.51]	< 0.001

The monthly prevalence was lowest at 4.08 per 1000 youths in January 2017, with the highest prevalence at 5.12 per 1000 youths in December 2021. Monthly prevalence rates of 7–12 year-old children showed a statistically significant decrease in the total group, from 5.10 (95% CI 5.00–5.20) per 1000 in the pre-COVID-19 period to 4.80 (95% CI 4.70–4.90) per 1000 during COVID-19. By contrast, the monthly prevalence rates increased, in girls between 13 and 19 years old, with an increase of 1.13 per 1000 youths. Overall, boys were more likely to use antipsychotic drugs.

**Fig. 2:**
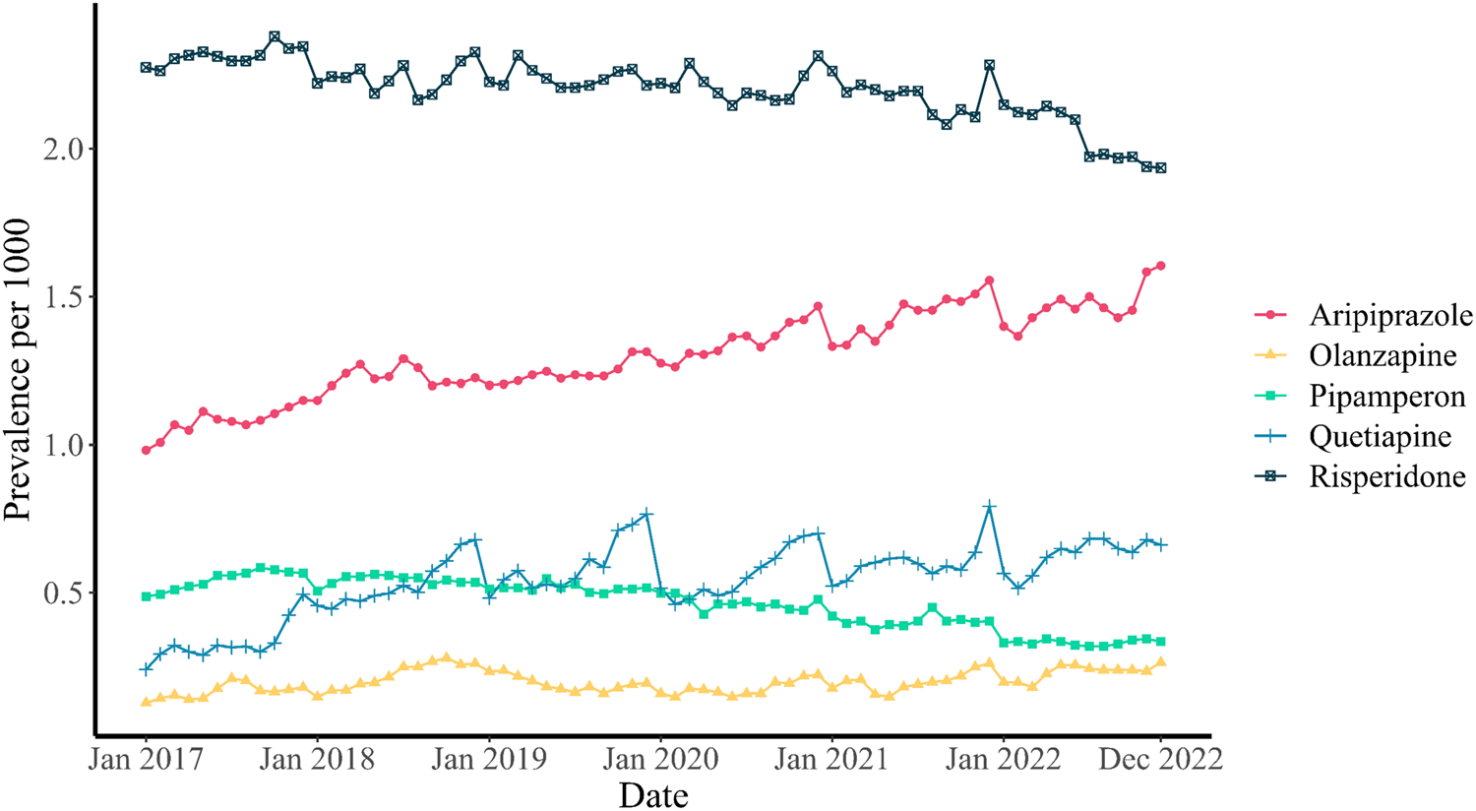
Monthly prevalence rate over the years stratified over the most prescribed antipsychotics

The most commonly prescribed antipsychotic drugs were Risperidone, Aripiprazole, Quetiapine, Pipamperone and Olanzapine (Fig. 2). In total, these antipsychotic drugs accounted for almost 96% of all prescribed antipsychotic drugs. Risperidone was the most frequently prescribed antipsychotic drug in all years with average monthly prevalence rates per 1000 youths around 2.26 pre-COVID-19 to 2.14 during COVID-19 (Supplementary Table 1). Aripiprazole and Quetiapine prescriptions increased during the COVID-19 period, while the prevalence rate of Pipamperon and Risperidone decreased compared to the pre-COVID-19 period. When the data were stratified by sex, males showed a statistically significant decrease in the prevalence rate for Pipamperon, and Risperidone users, while there was a significant increase in the prevalence rate for Aripiprazole users. In contrast, females showed an increase in the prevalence rate for Olanzapine, Aripiprazole, and Quetiapine users, and a decrease in the prevalence rate for Pipamperon users.

### Incidence

**Fig. 3:**
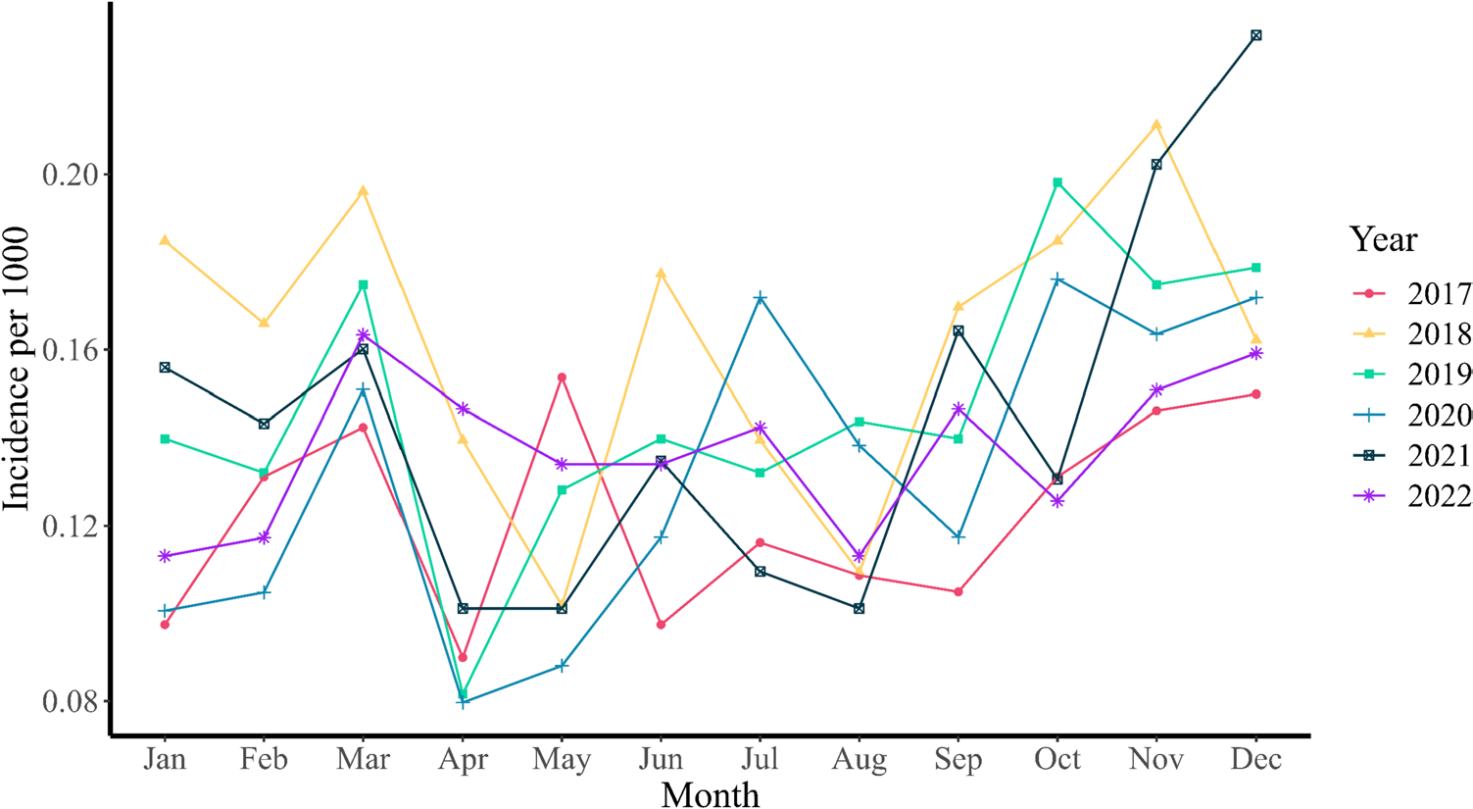
Monthly incidence rate over the years

The average monthly incidence rates of antipsychotic drug prescriptions among youths were consistent before and during the COVID-19 pandemic. Specifically, the rates were 0.14 (95% CI 0.13–0.15) pre-COVID-19 and 0.14 (95% CI 0.13–0.15) per 1000 youths during COVID-19. The incidence rates varied across time periods, with the lowest rate occurring in April 2020 at 0.075 per 1000 youths and the highest rate in December 2021 at 0.244 per 1000 youths. The incidence rates, stratified by age, sex and period, are presented in Table 2 and the monthly incidence rates over the years can be seen in Fig. 3. Overall, there was no significant increase in new antipsychotic drug prescriptions during these two periods. However, there was an increase in girls between the ages of 13 and 19 who started using these drugs during COVID-19 compared to pre-COVID-19.

**Table 2:** Average monthly incidence (per 1000) of antipsychotic drug prescriptions among Dutch youth up to 19 years

	Pre-COVID-19 (Jan 2017–Feb 2020)	COVID-19 (Mar 2020–Dec 2022)	*P*-value
Total			
All ages	0.14 [0.13, 0.15]	0.14 [0.13, 0.15]	0.911
0–6	0.01 [0.01, 0.01]	0.01 [0.01, 0.01]	0.106
7–12	0.05 [0.04, 0.05]	0.04 [0.03, 0.04]	0.036
13–19	0.09 [0.08, 0.10]	0.10 [0.09, 0.11]	0.186
Males			
All ages	0.16 [0.15, 0.18]	0.13 [0.12, 0.14]	0.003
0–6	0.02 [0.01, 0.02]	0.02 [0.01, 0.02]	0.516
7–12	0.07 [0.06, 0.08]	0.05 [0.05, 0.06]	0.010
13–19	0.08 [0.07, 0.09]	0.07 [0.06, 0.08]	0.060
Females			
All ages	0.12 [0.10, 0.13]	0.15 [0.13, 0.17]	0.005
0–6	0.01 [0.01, 0.01]	0.01 [0.01, 0.01]	0.444
7–12	0.02 [0.02, 0.03]	0.03 [0.02, 0.03]	0.879
13–19	0.09 [0.08, 0.11]	0.13 [0.11, 0.14]	0.001

The incidence rates of some antipsychotic drugs increased during the COVID-19 pandemic. Specifically, Aripiprazole and Olanzapine showed a slight increase, while Risperidone showed a slight decrease. These changes were statistically significant, although not very large in magnitude (Supplementary Table 2). When the data were stratified by sex, males showed a decrease in the incidence rate for Risperidone prescriptions.

### ARIMA model

**Fig. 4:**
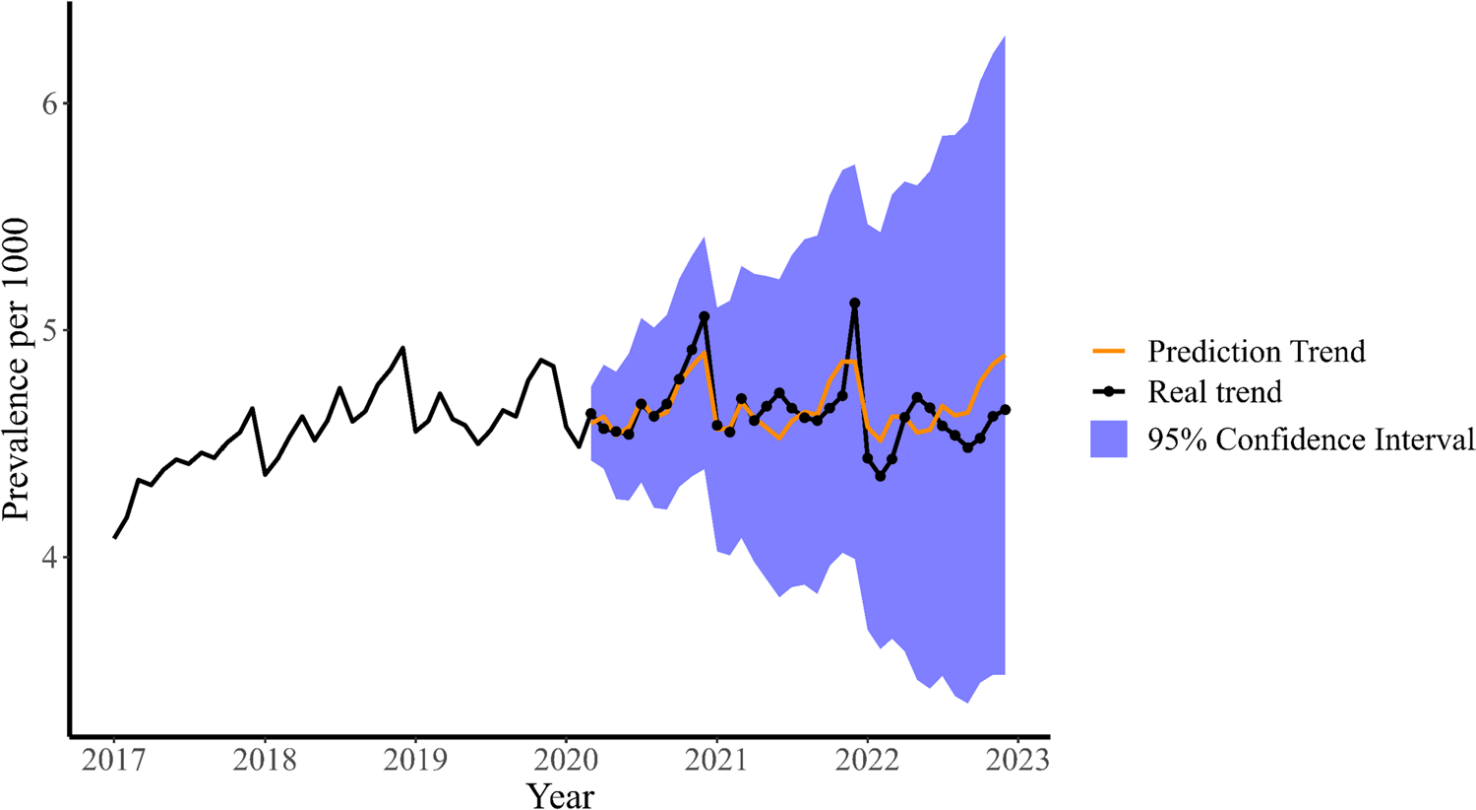
The ARIMA model of the monthly prevalence rate

We used the ARIMA model to forecast the counterfactual scenario of no COVID-19 based on the data from the pre-pandemic period. Figure 4 shows the result of this prediction. The orange line closely follows the dotted real trend, suggesting that there would be a rise in prevalence even without COVID-19. The MAPE value of 2.05% confirms that the model has a moderate error rate. Moreover, the real trend falls within the confidence bounds of the model, indicating that it is within our expectation and not statistically significant. The same holds for the monthly incidence (Supplementary Fig. 1).

#### Prescribers

**Fig. 5:**
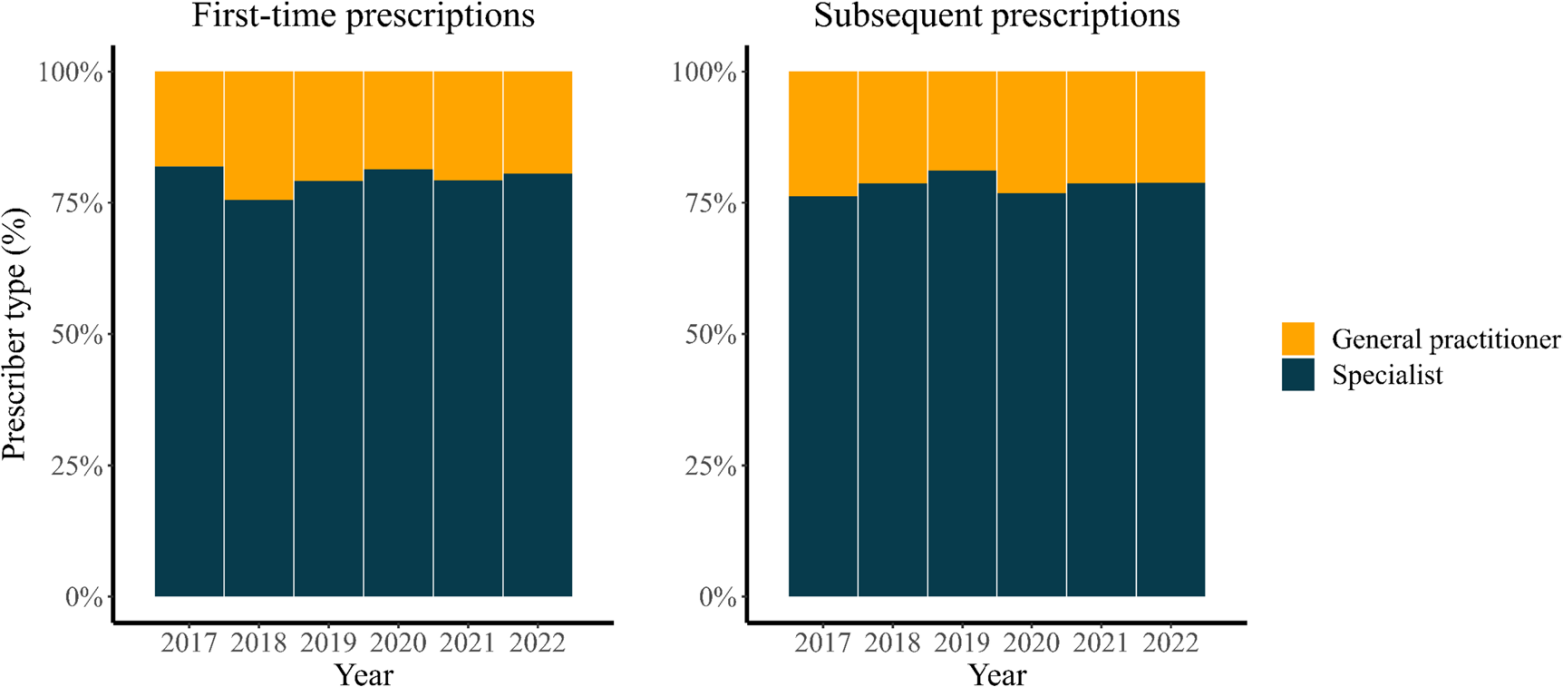
The proportion of prescriptions issued by general practitioners and specialists over the years

Figure 5 illustrates the distribution of the prescribers before and during the COVID-19 pandemic. GPs were responsible for 21% of both the initial and the follow-up prescriptions before the pandemic, with confidence intervals of [0.18, 0.25] and [0.19, 0.24], respectively. The remaining 79% were prescribed by specialists, with confidence intervals of [0.75, 0.82] and [0.76, 0.81], respectively. During the pandemic, the percentage of initial prescriptions from GPs slightly decreased to 20% [0.19, 0.21], while the percentage of follow-up prescriptions slightly increased to 22% [0.21, 0.23]. Specialists prescribed 80% [0.79, 0.81] of the initial prescriptions and 78% [0.77, 0.79] of the follow-up prescriptions during the pandemic. These differences between the two periods where not statistically significant.

#### Combined pharmacotherapy of antipsychotics

The analysis revealed that the prevalence of combined pharmacotherapy with antipsychotics did not change significantly during the pandemic. Before the pandemic, on average, about 0.25 [0.23, 0.26] per 1000 children received more than one antipsychotic in a year. During the pandemic, this number was about 0.20 [0.15, 0.29] per 1000 children. The difference was not statistically significant. The most frequent combination of antipsychotics was Aripiprazole and Risperidone.

## Discussion

In this study, where the reasons for prescribing antipsychotic are not known, our analysis revealed that antipsychotic prescription rates showed a moderate increase over time, and also during the COVID-19 period. This increase is consistent with the expected trend based on the ARIMA model, which was built using pre-pandemic data. Therefore, we cannot attribute the increase in antipsychotic use to the pandemic, as this rise would likely occur even without the pandemic. The monthly prevalence in the pre-pandemic period fluctuated around 4.56 user per 1000 youths and during the pandemic around 4.64 users per 1000 youths, with the highest number of users in December 2021 (5.11 per 1000 youths). During the pandemic, the monthly incidence rate of antipsychotic use did not change overall, but it increased among adolescent girls aged 13–19. Among the five most common antipsychotics, Aripiprazole and Quetiapine had higher monthly prevalence rates during the pandemic, while Risperidone had a lower rate. The monthly incidence rate rose for Aripiprazole and Olanzapine, and decreased for Risperidone during the COVID-19 pandemic.

The prevalence rate of antipsychotics increased over time and during the pandemic, with the same increase also seen in Denmark [13]. A possible explanation could be social isolation and heightened stress and anxiety levels [16, 17]. The peak of antipsychotic prevalence occurred in December 2021, coinciding with a lockdown in the Netherlands. The lockdowns caused more severe anxiety and sleep problems among the Dutch youth, which may have led to more prescriptions of antipsychotics [9]. We also examined the ARIMA model, which relied on the monthly prevalence rate of antipsychotic prescription prior to the pandemic. Our analysis revealed that despite the pandemic, the monthly moderate increase of the prevalence rate of antipsychotic prescription was within expectation, as can be seen in Fig. 4. This suggests that factors, beyond the pandemic, may be contributing to the rise in antipsychotic prescription in Dutch youth.

The most significant increase in antipsychotic prescriptions occurred among adolescent females aged 13–19 years. This group is particularly vulnerable to developing psychiatric disorders, such as anxiety, and depression, especially during adolescence [18, 19]. Additionally, they face a high risk of substance abuse and suicidal behavior, which may have worsened due to the disruption of school routines during the pandemic [20]. Lastly, social deprivation or isolation may have also contributed to this increase [17].

Moreover, both the monthly incidence and prevalence of antipsychotic prescriptions rose for adolescent females aged 13–19 years during the pandemic. This trend aligns with studies from the US and Canada, that reported a similar increase in psychotropic drug prescription for this age group [12, 21]. The main driver of this increase was the higher prescription rate of Quetiapine for adolescent females. They accounted for 55.95% of all Quetiapine prescriptions before the pandemic, but this rose to 69.05% during the pandemic. Quetiapine was often prescribed off-label to females with anxiety, insomnia, or depression before the pandemic [22, 23]. This off-label use in girls seems to have intensified during the pandemic, possibly due to the surge of mental health issues such as anxiety and depression in girls [8].

The monthly prevalence rate of Aripiprazole also increased during the pandemic. The pandemic may have affected the prescribing patterns of some healthcare providers, who opted for Aripiprazole over other medications for their patients, as they may have deemed it safer than the alternatives. The increase in Aripiprazole prescriptions during the pandemic could also be attributed to its availability and suitability for different patient populations. However, the claim that Aripiprazole is less harmful can be questioned, as there are contrasting results [24, 25].

Furthermore, we see a decrease in Pipamperon use during the pandemic. This first-generation antipsychotic drug is mainly used in the Netherlands and some other European countries for the treatment of psychoses and severe forms of excitement and agitation [26, 27]. However, the decline in Pipamperon use started before the pandemic, as reported by Bais et al. [14]. We speculate that this may be due to a lack of evidence on its effectiveness and safety [27], and a preference for more evidence-based treatments for mental health problems.

The combined pharmacotherapy of antipsychotics did not change significantly during the pandemic period. There is no clear evidence that combined pharmacotherapy of antipsychotics is safe or effective [28, 29]. The most frequent combination of antipsychotics before and during the pandemic was Aripiprazole together with Risperidone. This may be explained by the ability of aripiprazole to counteract hyperprolactinemia and its consequences caused by risperidone [30].

Finally, we examined whether the distribution of the prescribers of antipsychotics changed over time. We found that there was no significant change in the proportion of first-time and subsequent antipsychotic prescriptions issued by different types of providers during the pandemic. Specialists remained the main prescribers of both types of prescriptions, which is consistent with the prevailing treatment approach for these medications [31].

This paper explores how the prescription patterns of antipsychotic drugs for children and adolescents in the Netherlands changed before and during the COVID-19 pandemic. The increase of antipsychotic prescription, especially in adolescents females during the pandemic, is worrying and cannot only be attributed to the pandemic. With the off-label use of antipsychotics as one of the main reasons for the increased prescription rate in girls. We suggest that future research should examine other possible influences on these patterns, such as the socioeconomic status of the patients, their family environment and their quality of life.

### Strengths and limitations

The strength of this study is the use of IADB database, which has been shown to be representative of the national population of the Netherlands [32]. One limitation that should be taken into account when interpreting the results, is the lack of information on the reasons for prescribing medications, which could help explain the changes observed. Another limitation is that prescription rates do not reflect actual usage rates. We also did not have information on how well the patients adhered to their treatment.

## Conclusion

The main finding of this study was a moderate rise in antipsychotic prescription among Dutch youth over time and during the COVID-19 pandemic. Adolescent females aged 13–19 years showed a particularly notable increase in antipsychotic use. However, the study also suggests that factors beyond the pandemic may be contributing to the rise in antipsychotic prescription in Dutch youth. The study’s strength lies in the use of a representative database, but limitations include a lack of data on the reasons for prescribing medications and actual usage rates, as well as patient adherence to treatment. Further research is needed to better understand the factors driving the increase in antipsychotic prescription among Dutch youth and to inform appropriate clinical decision-making.

### Supplementary Information

Below is the link to the electronic supplementary material.

Supplementary file1 (DOCX 76 KB)

## Data Availability

The data that support the findings of this study are available in the database IADB.nl. However, these data are only made available after approval of a study protocol.

## Code availability

Syntaxes used to analyze the data can be made available upon reasonable request.
