# Correction to: Comparison of antipsychotic drug use in children and adolescents in the Netherlands before and during the COVID-19 pandemic

**DOI:** 10.1007/s00787-024-02434-6

**Published:** 2024-04-20

**Authors:** Ravish N. Gangapersad, Guiling Zhou, Pilar Garcia-Gomez, Jens Bos, Eelko Hak, Birgit C. P. Koch, Catharina C. M. Schuiling-Veninga, Bram Dierckx

**Affiliations:** 1 Department of Hospital Pharmacy, Erasmus University Medical Center, Rotterdam, The Netherlands; 2 Erasmus School of Economics, Erasmus University, Rotterdam, The Netherlands; 3 Unit of Pharmaco?Therapy, ?Epidemiology and ?Economics (PTEE), Department of Pharmacy, University of Groningen, Groningen, The Netherlands; 4 Department of Child and Adolescent Psychiatry/Psychology, Erasmus Medical Center, Rotterdam, The Netherlands

**Correction to: European Child & Adolescent Psychiatry**

10.1007/s00787-023-02340-3

In this article, the eighth author name Bram Dierckx, incorrectly published as Dierkcx .

The original article has been corrected.

