# Supplementary material for: Comparison of antipsychotic drug use in children and adolescents in the Netherlands before and during the COVID-19 pandemic
Source: Eur Child Adolesc Psychiatry. 2024 Jan 6;33(8):2695–703. doi: 10.1007/s00787-023-02340-3 (PMC11272724; doi:10.1007/s00787-023-02340-3)

# **Supplementary tables and figures**

**Table 1** Average monthly prevalence of the top five antipsychotics

|  | Pre-COVID-19 (Jan 2017 - Feb 2020) | COVID-19 (Mar 2020 - Dec 2022) | P-value |
| --- | --- | --- | --- |
| *Total* |  |  |  |
| *Aripiprazole* | 1.19 [1.16, 1.21] | 1.43 [1.40, 1.45] | <0.001 |
| *Olanzapine* | 0.19 [0.18, 0.20] | 0.20 [0.19, 0.22] | 0.115 |
| *Pipamperon* | 0.53 [0.52, 0.54] | 0.39 [0.38, 0.41] | <0.001 |
| *Quetiapine* | 0.49 [0.44, 0.53] | 0.61 [0.58, 0.63] | <0.001 |
| *Risperidone* | 2.26 [2.24, 2.28] | 2.14 [2.11, 2.18] | <0.001 |
| *Males* |  |  |  |
| *Aripiprazole* | 0.84 [0.83, 0.86] | 0.97 [0.96, 0.98] | <0.001 |
| *Olanzapine* | 0.09 [0.08, 0.10] | 0.08 [0.08, 0.09] | 0.13 |
| *Pipamperon* | 0.36 [0.36, 0.37] | 0.26 [0.24, 0.27] | <0.001 |
| *Quetiapine* | 0.20 [0.19, 0.22] | 0.19 [0.18, 0.20] | 0.060 |
| *Risperidone* | 1.71 [1.70, 1.73] | 1.60 [1.56, 1.63] | <0.001 |
| *Females* |  |  |  |
| *Aripiprazole* | 0.34 [0.33, 0.36] | 0.45 [0.44, 0.47] | <0.001 |
| *Olanzapine* | 0.10 [0.10, 0.11] | 0.12 [0.12, 0.13] | <0.001 |
| *Pipamperon* | 0.17 [0.16, 0.17] | 0.14 [0.13, 0.14] | <0.001 |
| *Quetiapine* | 0.28 [0.25, 0.31] | 0.42 [0.40, 0.44] | <0.001 |
| *Risperidone* | 0.55 [0.54, 0.56] | 0.55 [0.53, 0.56] | 0.807 |

**Table 2** Average monthly incidence of the top five antipsychotics

|  | Pre-COVID-19 (Jan 2017 – Feb 2020) | COVID-19 (Mar 2020 - Dec 2022) | P-value |
| --- | --- | --- | --- |
| *Total* |  |  |  |
| *Aripiprazole* | 0.04 [0.04, 0.05] | 0.06 [0.05, 0.06] | 0.04 |
| *Olanzapine* | 0.02 [0.02, 0.02] | 0.03 [0.02, 0.03] | 0.011 |
| *Pipamperon* | 0.02 [0.02, 0.02] | 0.02 [0.01, 0.02] | 0.293 |
| *Quetiapine* | 0.07 [0.06, 0.09] | 0.08 [0.07, 0.09] | 0.294 |
| *Risperidone* | 0.13 [0.12, 0.14] | 0.10 [0.09, 0.11] | 0.003 |
| *Males* |  |  |  |
| *Aripiprazole* | 0.03 [0.02, 0.03] | 0.03 [0.03, 0.04] | 0.303 |
| *Olanzapine* | 0.01 [0.01, 0.02] | 0.01 [0.01, 0.01] | 0.731 |
| *Pipamperon* | 0.01 [0.01, 0.02] | 0.01 [0.01, 0.02] | 0.918 |
| *Quetiapine* | 0.03 [0.02, 0.03] | 0.02 [0.02, 0.03] | 0.228 |
| *Risperidone* | 0.09 [0.08, 0.10] | 0.07 [0.06, 0.07] | <0.001 |
| *Females* |  |  |  |
| *Aripiprazole* | 0.02 [0.02, 0.03] | 0.03 [0.02, 0.03] | 0.057 |
| *Olanzapine* | 0.02 [0.01, 0.02] | 0.02 [0.02, 0.02] | 0.009 |
| *Pipamperon* | 0.01 [0.01, 0.01] | 0.01 [0.01, 0.01] | 0.295 |
| *Quetiapine* | 0.05 [0.04, 0.06] | 0.06 [0.05, 0.07] | 0.053 |
| *Risperidone* | 0.04 [0.03, 0.04] | 0.04 [0.03, 0.04] | 0.946 |

**Table 3**: The distribution of the population over the different age groups.

|  | Total (%) | Male (%) | Female (%) |
| --- | --- | --- | --- |
| 0-6 | 30 | 16 | 14 |
| 7-12 | 29 | 15 | 14 |
| 13-19 | 41 | 20 | 21 |

**Fig 1.** The ARIMA model of the monthly incidence rate.
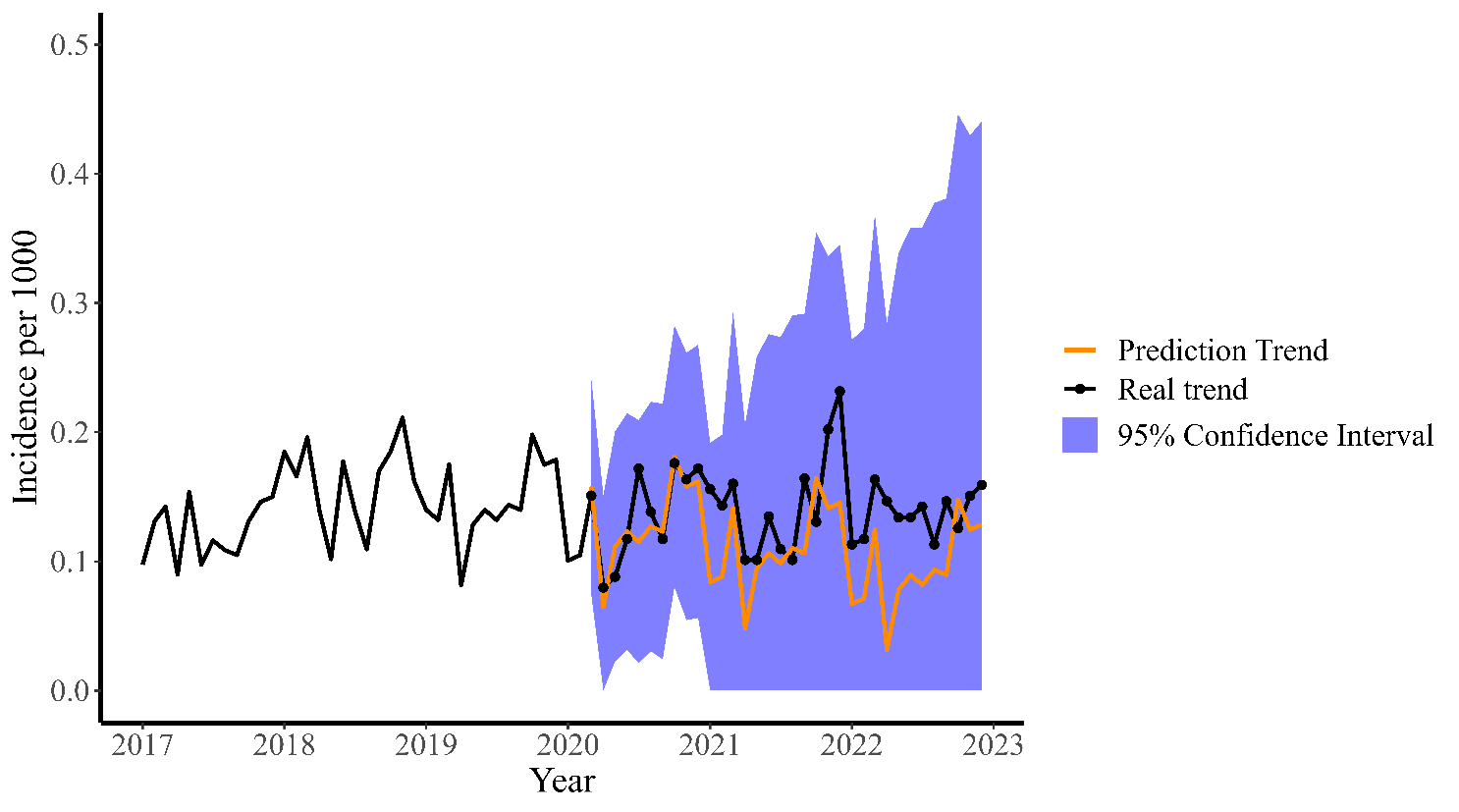

Supplement: Supplementary file 1 — Supplementary file1 (DOCX 76 KB) [file 787_2023_2340_MOESM1_ESM.docx]
